# Human papillomavirus vaccine effect against human papillomavirus infection in Rwanda: evidence from repeated cross-sectional cervical-cell-based surveys

**DOI:** 10.1016/S2214-109X(23)00193-6

**Published:** 2023-05-16

**Authors:** Felix Sayinzoga, Vanessa Tenet, Daniëlle A M Heideman, Hassan Sibomana, Marie-Chantal Umulisa, Silvia Franceschi, Jean de Dieu Hakizimana, Gary M Clifford, Iacopo Baussano

**Affiliations:** a Ministry of Health, Rwanda Biomedical Center, Kigali, Rwanda; b Susan Thompson Buffett Foundation, Kigali, Rwanda; c Early Detection, Prevention and Infections Branch, International Agency for Research on Cancer (IARC/WHO), Lyon, France; d Pathology, Amsterdam UMC, location Vrije Universiteit Amsterdam, Amsterdam, Netherlands; e Cancer Center Amsterdam, Imaging and Biomarkers, Amsterdam, Netherlands; f Centro di Riferimento Oncologico (CRO), IRCCS, Aviano, Italy

## Abstract

**Background:**

Rwanda was the first African country to implement national human papillomavirus (HPV) vaccination (against types HPV6, 11, 16, and 18). In 2011, a school-based catch-up programme was initiated to vaccinate girls aged younger than 15 years but it also reached older girls in schools. We aimed to estimate the population-level effect of HPV vaccination on HPV prevalence.

**Methods:**

Cross-sectional surveys were done between July, 2013, and April, 2014 (baseline), and between March, 2019, and December, 2020 (repeat), in sexually active women aged 17–29 years at health centres in the Nyarugenge District of Kigali, Rwanda. HPV prevalence was assessed in cervical cell samples collected by a health-care worker in PreservCyt solution (Cytyc, Boxbourough, MA, USA) and tested using a general primer (GP5+ or GP6+)-mediated PCR. Adjusted overall, total, and indirect (herd immunity) vaccine effectiveness was computed as the percentage of HPV detection among all women and among unvaccinated women.

**Findings:**

1501 participants completed the baseline survey and 1639 completed the repeat survey. HPV vaccine-type prevalence in participants aged 17–29 years decreased from 12% (173 of 1501) in the baseline survey to 5% (89 of 1639) in the repeat survey, with an adjusted overall vaccine effectiveness of 47% (95% CI 31 to 60) and an adjusted indirect vaccine effectiveness of 32% (9 to 49). Among participants aged 17–23 years, who were eligible for catch-up vaccination, the adjusted overall vaccine effectiveness was 52% (35 to 65) and the adjusted indirect vaccine effectiveness was 36% (8 to 55), with important heterogeneity according to education (overall vaccine effectiveness was 68% [51 to 79] in participants with ≥6 years of school completed and 16% [–34 to 47] in those with <6 years) and HIV status (overall vaccine effectiveness was 55% [36 to 69] for HIV-negative participants and 24% [–62 to 64] for HIV-positive participants).

**Interpretation:**

In Rwanda, the prevalence of vaccine-targeted HPV types has been significantly decreased by the HPV vaccine programme, most notably in women who were attending school during the catch-up programme in 2011. HPV vaccine coverage and population-level impact is expected to increase in future cohorts who are eligible for routine HPV vaccination at age 12 years.

**Funding:**

Bill & Melinda Gates Foundation.

## Introduction

In Rwanda, cervical cancer is the second most common cancer among women, with an age-standardised (on the world population) incidence rate of 28 cases per 100 000 population,^1^ and in 2011 the country was the first African nation to implement a national human papillomavirus (HPV) vaccination programme to protect girls aged younger than 15 years (ie, born since 1997). After an initial national school-based catch-up campaign in 2011, there was a transition to routine vaccination of girls aged 12 years, all with a three-dose schedule of vaccine targeting types HPV6, 11, 16, and 18.^2^ The school-based catch-up campaign resulted in some vaccination coverage gaps, which were likely to be explained by out-of-school teenagers. However, the programme also reached many girls aged 15 years or older, whose schooling had been delayed and who were hence still studying in the eligible school grades. Vaccination coverage was high in all cohorts who were routinely offered the vaccine at age 12 years (80−90%).^3^ Since 2015, the programme follows a two-dose vaccination schedule.

The Ministry of Health of Rwanda and the International Agency for Research on Cancer (IARC) have collaborated for 10 years on a series of cross-sectional surveys to show the real-life effect of HPV vaccination at a population level. HPV prevalence was assessed in surveys involving urine samples among girls in schools^4^ and in surveys involving cervical cell samples among the sexually active general female population.^5^ The results of the baseline cervical-cell-based survey conducted in the Nyarugenge District were published in 2016.^5^ In this Article, we estimate the population-level effect of HPV vaccination on HPV prevalence by comparing HPV prevalence in baseline and repeated surveys, overall and according to population strata (most notably age, schooling history, and HIV status). We also estimate the number of future cervical cancer cases in Rwanda in vaccine-targeted birth cohorts (born in 1997 or later) by vaccination status.

**Research in context**

**Evidence before this study**

The favourable impact of human papillomavirus (HPV) vaccination on HPV burden and cervical cancer incidence at a population level has been repeatedly shown in high-income settings. We searched PubMed on Jan 1, 2023, using the terms “HPV”, “vaccine”, and “epidemiology”, with no language or date restrictions, to identify studies reporting the biological effect of HPV vaccination at a population level in low-income or middle-income countries. A series of cross-sectional surveys in Bhutan, a middle-income country where vaccination coverage reached the target recommended by WHO for cervical cancer elimination as a public health problem (ie, 90%), showed that infections with vaccine-targeted HPV types can be almost eliminated among birth cohorts with a high vaccination coverage. In low-income settings, the only empirical assessment of HPV vaccination effect is from Rwanda, through a series of urine-based surveys among girls in schools. A report from 2020 in Rwanda found national coverage of HPV vaccination in birth cohorts who were offered the HPV vaccine in the catch-up programme to be significantly lower than that in birth cohorts who were offered routine vaccination at age 12 years. Based on this finding, it is reasonable to expect different levels of effect of HPV vaccination in different birth cohorts in Rwanda. However, to our knowledge, no empirical assessment of the effect of HPV vaccination at a population level has yet been reported in Rwanda, in Africa, or in any other low-income setting.

**Added value of this study**

In this study, we aimed to estimate the effect of HPV vaccination on HPV prevalence in Rwanda and on future cervical cancer incidence, based on empirical data collected in a series of cross-sectional surveys in the general population. The self-reported vaccine coverage among study participants eligible for catch-up HPV vaccination was shown to be significantly lower than the 90% target recommended by WHO. Furthermore, HPV vaccination coverage, and consequently the effect of HPV vaccine on HPV prevalence, was shown to differ with respect to level of education and HIV status.

**Implications of all the available evidence**

Quantifying the effect of HPV vaccination and its heterogeneity across population subgroups in Rwanda is likely to have implications both locally and on a wider scale. Based on our findings, Rwandan public health authorities can reinforce their political and financial commitment towards cervical cancer elimination, improve vaccination coverage, and design adapted cervical cancer screening strategies. Public health authorities in other countries in sub-Saharan Africa and elsewhere can learn from the Rwandan example, both in terms of implementing HPV vaccination and of monitoring its impact on HPV prevalence and cervical cancer burden.

## Methods

### Study design

**Figure 1:**
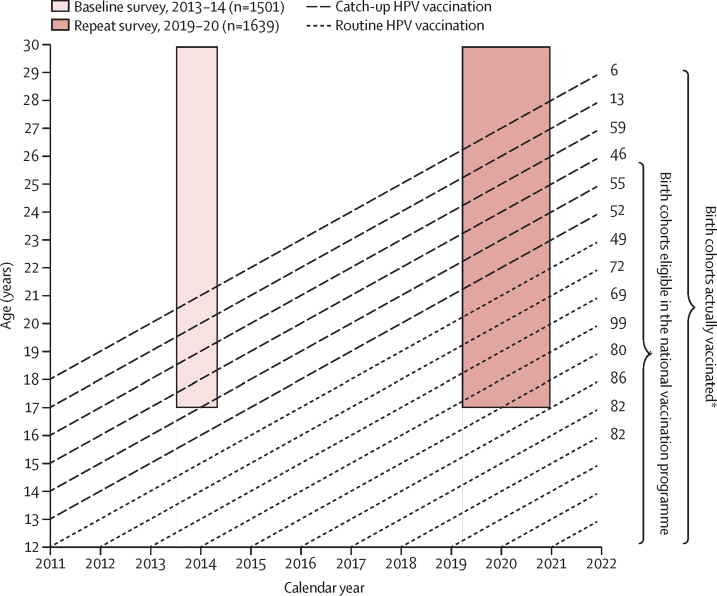
Timeline for school-based HPV vaccination programme and surveys in Rwanda Long-dashed lines represent birth cohorts vaccinated during the catch-up campaign; short-dashed lines represent birth cohorts vaccinated through routine vaccination. HPV=human papillomavirus. *As reported by Sayinzoga and colleagues.^3^

Cross-sectional surveys were done between July, 2013, and April, 2014 (baseline), and between March, 2019, and December, 2020 (repeat), in sexually active women aged 17–29 years at health centres in the Nyarugenge District of Kigali, Rwanda. This age range was selected to focus on birth cohorts who were potentially covered by the national HPV vaccination programme in the repeat survey. The vaccination programme began in 2011 but, as girls up to age 15 years in school grades P6 (last year of primary school) and S3 (last year of lower secondary school) were included,^2^ 99% of women recruited in the baseline survey were unvaccinated.^5^ In the repeat survey, women could have been vaccinated if they were in grades P6 or S3 during the catch-up vaccination programme in 2012–14 (figure 1).

Baseline survey procedures for study population recruitment, collection of cervical cells, DNA extraction, HPV testing, and genotyping have been previously reported.^5^ To enable comparison of prevalence estimates over time, we used the same procedures in the repeat survey.

This study was approved by the Rwanda National Ethical Committee of the Ministry of Health of Rwanda (480/RNEC/2013, 457/RNEC/2018, and 751/RNEC/2020) and the IARC Ethics Committee (13-06 and 13-07).

### Participants

In the repeat survey, we aimed to recruit around 1500 sexually active women aged 17–29 years and to match, as far as possible, the distribution by age and catchment areas studied in the baseline survey. Women were recruited in nine health centres in the baseline survey and 11 health centres in the repeat survey (appendix p 5). Women attending outpatient clinics or accepting invitations from community health-care workers were included. We have previously reported the method used to define the size of the surveys.^6^ In brief, based on findings from cross-sectional surveys previously conducted by IARC in other sub-Saharan African countries, we hypothesised that the prevalence of HPV6, 11, 16, and 18 (ie, HPV types targeted by the vaccine) could range between 7% and 9%. We then defined the size of the surveys to be able to detect a vaccine-induced HPV prevalence reduction in the target age range of at least 50% (α error of 5% and β error of 10%). All study participants gave consent by signing an informed consent form (appendix p 13).

### Procedures

Using a predefined questionnaire (appendix p 15), up to two trained health-care workers affiliated to each health-care centre conducted an interview to collect data from participants on sociodemographic characteristics, sexual history, and self-reported HPV vaccination status (including number of doses and age at first dose). The same health-care workers also collected cervical cell samples. Data were regularly transferred to IARC via a specifically designed online application that cross-checked for inconsistencies. At the same visit, participants had a cervical cell collection procedure performed by a health-care worker. Both a completed questionnaire and a cervical cell sample were required for inclusion in the study. Cytology samples and questionnaires could be matched via an anonymised identification number that was known only to the local principal investigator (JdDH). Exfoliated cervical cells were collected from the endocervix and ectocervix using a cytobrush (Cervex-Brush; Rovers Medical Devices, Oss, Netherlands), then placed in a vial containing PreservCyt solution (Cytyc, Boxbourough, MA, USA) and stored at 4°C until shipment. As in the baseline survey, HPV testing was conducted at the pathology department of Amsterdam UMC (location Vrije Universiteit Amsterdam, Amsterdam, Netherlands), where DNA was extracted using magnetic beads (NucleoMag 96 kit; Macherey-Nagel, Düren, Germany) on a Microlab Star robotic system (Hamilton Robotics, Reno, NV, USA) following the manufacturer's instructions. The presence of human DNA in the specimens was assessed by β-globin PCR^7^ and presence of HPV DNA was assessed by a general primer (GP5+ or GP6+)-mediated PCR, which detects a broad spectrum of genital HPV types. To assess HPV positivity, PCR products were hybridised in an enzyme immunoassay with two oligonucleotide probe cocktails jointly capable of detecting 44 mucosal HPV types, including HPV vaccine-taregeted types (HPV6, 11, 16, and 18), other alpha-9 types (HPV31, 33, 35, 52, and 58), other alpha-7 types (HPV39, 45, 59, and 68), and 31 non-alpha-7 and non-alpha-9 types (HPV51, 56, 26, 30, 32, 34, 40, 42, 43, 44, 53, 54, 55, 57, 61, 64, 66, 67, 69, 70, 71, 72, 73, 81, 82, 83, 84, 85, 86, 89, and 90).^8^ Samples that were positive on enzyme immunoassay were HPV genotyped by reverse-line blot or Luminex-based hybridisation of GP5+ or GP6+ PCR products. Results were considered invalid when no HPV nor β-globin was detectable.

### Statistical analysis

We described selected characteristics of the women included in the baseline and repeat surveys. Type-specific HPV prevalence was assessed in both surveys by grouping HPV types as vaccine types or non-vaccine types. To assess for cross-protection attributable to vaccination, non-vaccine types were further stratified into other alpha-9 types, other alpha-7 types, and all other detectable non-alpha-7 and non-alpha-9 types.

We estimated the effect of HPV vaccination at a population level in Rwanda using an adaptation of the method proposed by Halloran and colleagues^9^ that includes different types of effectiveness by comparing different groups of women based on self-reported vaccination status (appendix p 10). We compared type-specific HPV prevalence in: all women in the baseline and repeat surveys to estimate overall effectiveness (a measure of the effect of vaccination at a population level); unvaccinated women in the baseline survey and vaccinated women in the repeat survey to estimate total effectiveness (a measure of the effect of vaccination among vaccinated women in the population); and unvaccinated women in the baseline and repeat surveys to estimate indirect effectiveness (a measure of the effect of vaccination among unvaccinated women in the population—ie, herd effect). In the base case, women who were unsure about their vaccination status were considered to be unvaccinated, whereas in the sensitivity analyses for total and indirect effectiveness, these women were alternatively excluded or considered to be vaccinated. Of note, estimates of overall effectiveness were not affected by the reported vaccination status of the women or its misclassification. Finally, to ensure that differences in health centres across the two surveys did not materially affect our estimates, we did a sensitivity analysis restricted to the nine health centres participating in both surveys, with health centre as a clustering variable.

We estimated vaccine effectiveness as complementary to HPV prevalence ratios (PRs). HPV PRs and corresponding 95% CIs were estimated using binomial regression models with a log link. PR estimates were adjusted for respondent's age group (ie, 17–23 years or 24–29 years), HIV status (negative, positive, or unknown), years of education completed (<6 or ≥6), and lifetime number of sexual partners (ie, 1, 2, or ≥3), to account for any demographic or behavioural changes between the two surveys. The adjusted PRs were used to estimate adjusted HPV vaccine effectiveness (1 – PR) and their corresponding 95% CIs.

Overall, total, and indirect vaccine effectiveness percentages were estimated for all women aged 17−29 years, and separately for those who were originally eligible for the vaccine in the national HPV vaccination programme (ie, age younger than 24 years *vs* age 24−29 years). We also estimated vaccine effectiveness stratified by years of education and HIV status. Statistical analyses were done using Stata SE (version 15.1). Adjusted PRs were assessed using the “glm, fam(bin) link(log)” procedure. Observations with missing data were omitted from the analyses and no imputation of the missing data was attempted. Of note, in our analysis, there was no allowance for multiple testing.

Finally, based on the aforementioned estimates of the overall effectiveness of vaccination on HPV prevalence, we also projected the estimated effect of HPV vaccination on cervical cancer burden in Rwanda. We calculated the expected lifetime risk of cervical cancer and the annual age-standardised incidence rate, standardised on the world standard population, in birth cohorts of women aged 17–23 years in the baseline (without vaccination) and repeat (with vaccination) surveys. These projections were drawn under the assumption that the overall adjusted vaccine effectiveness estimated from our surveys was representative of the whole country and of lifelong HPV vaccination protection. The projections were performed with R software (version 4.1.2) using the method proposed by Bonjour and colleagues,^10^ described in the appendix (pp 2–3). In brief, the expected number of cervical cancer cases in the absence of vaccination was quantified by combining age-specific incidence rates from GLOBOCAN 2020 and cohort-specific mortality rates in Rwanda by age from UN demographic projections. Preventable cancers were estimated on the basis of HPV prevalence reduction attributable to vaccination and the relative contribution of each HPV type to cervical cancer incidence. We assessed the number of cervical cancer cases preventable through vaccines targeting HPV types 16 and 18, with cross-protection against HPV types 31, 33, and 45.

### Role of the funding source

The funder of the study had no role in study design, data collection, data analysis, data interpretation, writing of the report, or the decision to submit for publication.

## Results

In the baseline survey, 1509 women signed the informed consent form, 1503 had cervical cell collection, and 1501 (>99%) had a valid HPV test and completed the study questionnaire. In the repeat survey, 1689 women signed the informed consent form, 1685 had cervical cell collection, and 1639 (97%) had a valid HPV test and completed the study questionnaire (appendix p 11). Approximately 50% of all women were recruited in three of the 11 health centres (Rugarama, Kabusunzu, and Nyarurenzi; table 1). HPV vaccination was reported by 638 (39%) of 1639 eligible women, whereas 108 (7%) were unsure about their vaccination status. 369 (58%) of 638 women reported receiving the vaccine at age younger than 15 years, and 553 (87%) reported more than one dose. Median age at vaccination in the repeat survey was 14 years (IQR 13−15), whereas vaccinated participants in the baseline survey were older (median age 16 years [IQR 15−17]), more frequently born outside Kigali (62% [n=1020] *vs* 54% [n=815]), more frequently literate (90% [n=1514] *vs* 88% [n=1323]), and more frequently reported being housewives (64% [n=1042] *vs* 45% [n=678]). The proportion of women who reported being HIV-positive was 12% in both surveys. However, the proportion of women with an uncertain HIV status was lower in the repeat survey than in the baseline survey (2% *vs* 20%; table 1).

**Table 1:** Participant characteristics

	**Baseline survey (n=1501)**	**Repeat survey (n=1639)**
**Age, years**		
17–19	197 (13%)	230 (14%)
20–21	356 (24%)	529 (32%)
22–23	539 (36%)	432 (26%)
24–25	213 (14%)	166 (10%)
26–27	81 (5%)	130 (8%)
28–29	115 (8%)	152 (9%)
**Health centre**		
Muhima Hospital	232 (16%)	98 (6%)
Muhima	150 (10%)	37 (2%)
Butamwa	176 (12%)	119 (7%)
Rugarama	180 (12%)	334 (20%)
Mwendo	167 (11%)	110 (7%)
Cornum	190 (13%)	96 (6%)
Gitega	121 (8%)	111 (7%)
Kabusunzu	126 (8%)	313 (19%)
Biryogo	159 (11%)	127 (8%)
Kanyinya and Nzove	0	95 (6%)
Nyarurenzi	0	199 (12%)
**Place of birth**		
Kigali	686 (46%)	617 (38%)
Outside Kigali	815 (54%)	1020 (62%)
Missing	0	2 (<1%)
**Level of education (school years completed)**		
Non-literate (0–5)	178 (12%)	125 (8%)
Literate (0–5)	468 (31%)	394 (24%)
Literate (6–10)	665 (44%)	830 (51%)
Literate (≥11)	190 (13%)	256 (16%)
Missing	0	34 (2%)
**Occupation**		
Housewife	678 (45%)	1042 (64%)
Shopkeeper or saleperson	203 (14%)	138 (8%)
Manual worker	251 (17%)	167 (10%)
Teacher, health-care worker, student, or clerical staff	75 (5%)	94 (6%)
Farmer	285 (19%)	136 (8%)
Other	9 (1%)	59 (4%)
Missing	0	3 (<1%)
**Marital status**		
Married or cohabiting	1051 (70%)	1078 (66%)
Single	383 (26%)	471 (29%)
Separated or widowed	67 (5%)	83 (5%)
Missing	0	7 (<1%)
**Smoking**		
Never	1432 (95%)	1553 (95%)
Ever	68 (5%)	74 (5%)
Missing	1 (<1%)	12 (1%)
**Number of pregnancies**		
Nulliparous	167 (11%)	170 (10%)
1	741 (49%)	876 (53%)
2–3	540 (36%)	539 (33%)
≥4	50 (3%)	43 (3%)
Missing	3 (<1%)	11 (1%)
Lifetime sexual partners*		
1	690 (46%)	677 (42%)
2	408 (27%)	479 (29%)
3	145 (10%)	244 (15%)
≥4	134 (9%)	199 (12%)
Missing	121 (8%)	29 (2%)
Age at first sexual intercourse, years*		
≤18	861 (58%)	1016 (62%)
19–20	321 (21%)	338 (21%)
≥21	194 (13%)	194 (12%)
Missing	122 (8%)	80 (5%)
Sexual activity in the previous year*		
Yes	1429 (95%)	1527 (94%)
No	58 (4%)	60 (4%)
Missing	11 (1%)	41 (3%)
History of receiving cash for sex*		
No	1383 (92%)	1341 (82%)
Yes	103 (7%)	270 (17%)
Prefer not to answer	0	8 (<1%)
Missing	12 (1%)	9 (1%)
**HIV status**		
Negative	1026 (68%)	1413 (86%)
Positive	181 (12%)	190 (12%)
Unknown	294 (20%)	26 (2%)
Missing	0	10 (1%)
**HPV vaccination**		
No	1478 (99%)	871 (53%)
Yes	17 (1%)	638 (39%)
Not sure	0	108 (7%)
Missing	6 (<1%)	22 (1%)
**Number of vaccine doses**†		
1	7 (41%)	65 (10%)
2–3	10 (59%)	553 (87%)
Missing	0	20 (3%)
**Age at vaccination, years**‡		
<15	0	369 (58%)
≥15	16 (94%)	162 (25%)
Missing	1 (6%)	107 (17%)

Data are n (%). HPV=human papillomavirus.

* Three women in the baseline survey and 11 in the repeat survey had no previous sexual partners and were excluded.

† Among 17 women in the baseline survey and 618 in the repeat survey who were vaccinated and had dose available.

‡ Among 16 women in the baseline survey and 531 in the repeat survey who were vaccinated and had age at vaccination available.

**Figure 2:**
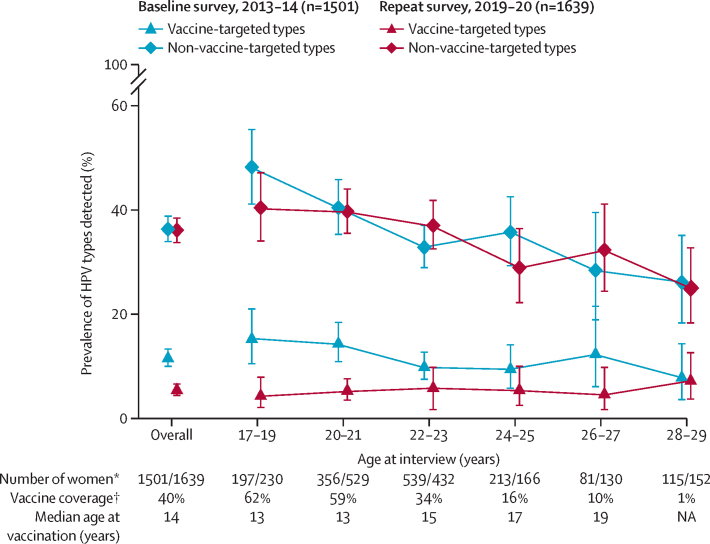
HPV prevalence in baseline and repeat surveys in Rwanda by HPV type and age Error bars show 95% CI. Vaccine-targeted types (HPV6, 11, 16, and 18); non-vaccine-targeted types (40 types detected by general primer [GP5+ or GP6+]-mediated PCR other than the four vaccine-targeted types). HPV=human papillomavirus. NA=not applicable. *Baseline survey/repeat survey. †Repeat survey.

Overall prevalence of HPV6, 11, 16, and 18 in women aged 17–29 years decreased from 12% (173 of 1501) in the baseline survey to 5% (89 of 1639) in the repeat survey (crude reduction of 53%, 95% CI 40–63; table 2, figure 2). The reduction was greatest in those aged 17–19 years (from 15% [30 of 197] in the baseline survey to 4% [ten of 230] in the repeat survey), in whom vaccine coverage was the highest (figure 2). Overall vaccine effectiveness was 47% (95% CI 31–60) among all women, and 52% (35–65) among those aged 17–23 years. No significant overall vaccine effectiveness was detected among women aged 24−29 years. Total vaccine effectiveness was 70% (95% CI 52–82) among all women, and 69% (50–81) among those aged 17–23 years. Indirect vaccine effectiveness was 32% (95% CI 9–49) among all women, and 36% (8–55) among those aged 17–23 years (table 2).

**Table 2:** HPV prevalence rates, prevalence ratios, and vaccine effectiveness by HPV type

		**Baseline survey**		**Repeat survey**		Adjusted vaccine effectiveness (95% CI)*
		Number assessed	HPV-positive	Number assessed	HPV-positive	
**HPV6, 11, 16, and 18**						
All ages						
	Overall†	1501	173 (12%)	1639	89 (5%)	47% (31 to 60)
	Indirect‡	1478	169 (11%)	979	69 (7%)	32% (9 to 49)
	Total§	1478	169 (11%)	638	20 (3%)	70% (52 to 82)
Age 17–23 years						
	Overall†	1092	134 (12%)	1191	63 (5%)	52% (35 to 65)
	Indirect‡	1072	130 (12%)	585	43 (7%)	36% (8 to 55)
	Total§	1072	130 (12%)	597	20 (3%)	69% (50 to 81)
Age 24–29 years						
	Overall†	409	39 (10%)	448	26 (6%)	37% (−5 to 62)
	Indirect‡	406	39 (10%)	394	26 (7%)	30% (−16 to 58)
	Total§	406	39 (10%)	41	0	100% (NA)
**Other alpha-9 HPV types**¶						
All ages						
	Overall†	1501	205 (14%)	1639	168 (10%)	28% (11 to 41)
	Indirect‡	1478	200 (14%)	979	105 (11%)	26% (6 to 42)
	Total§	1478	200 (14%)	638	63 (10%)	25% (−1 to 45)
Age 17–23 years						
	Overall†	1092	151 (14%)	1191	123 (10%)	28% (8 to 43)
	Indirect‡	1072	146 (14%)	585	64 (11%)	27% (2 to 46)
	Total§	1072	146 (14%)	597	59 (10%)	24% (−5 to 44)
Age 24–29 years						
	Overall†	409	54 (13%)	448	45 (10%)	26% (−10 to 50)
	Indirect‡	406	54 (13%)	394	41 (10%)	26% (−11 to 51)
	Total§	406	54 (13%)	41	4 (10%)	−9% (−145 to 66)

Data are n or n (%) unless otherwise stated. HPV=human papillomavirus. NA=not applicable.

* Adjusted for age, level of education, HIV status, and lifetime number of sexual partners.

† Entire baseline survey group compared with entire repeat survey group.

‡ Unvaccinated baseline survey group compared with entire repeat survey group.

§ Unvaccinated baseline survey group compared with vaccinated repeat survey group.

¶ Other alpha-9 types (HPV31, 33, 35, 52, and 58).

The prevalence of HPV types not targeted by the vaccine did not significantly vary between the two surveys in any age group (figure 2). Nevertheless, we detected a significant reduction in the prevalence of other alpha-9 HPV types from 14% (205 of 1501) in the baseline survey to 10% (168 of 1693) in the repeat survey among all women (overall vaccine effectiveness 28%, 95% CI 11−41), and from 14% (151 of 1092) in the baseline survey to 10% (123 of 1191) in the repeat survey among women aged 17–23 years (vaccine effectiveness 28%, 8−43; table 2). Corresponding indirect cross-protective vaccine effectiveness was 26% (95% CI 6−42) among all women and 27% (2−46) among women aged 17–23 years. No effect of vaccination on other alpha-7 HPV types and non-alpha-7 and non-alpha-9 types was detected in any age group (appendix p 6). In sensitivity analyses, when 108 (7%) women who were not sure about their vaccination status in the repeat survey were excluded or considered as vaccinated, there were no substantial changes in the total and indirect vaccine effectiveness estimates (appendix pp 7–8). Similarly, in analyses restricted to the nine health centres that participated in both surveys with health centres modelled as a clustering variable, the estimates of vaccine effectiveness were not materially different from those obtained in the primary analysis (appendix p 9).

In women aged 17–23 years who had completed at least 6 years of school, in whom vaccine coverage was 63% (median age at vaccination of 14 years, IQR 13–15), there was a significant reduction in HPV6, 11, 16, and 18 prevalence from 13% (95% CI 11 to 16) in the baseline survey to 4% (3 to 6) in the repeat survey, with an overall effectiveness of 68% (51 to 79; table 3). Conversely, among women aged 17–23 years who had completed less than 6 years of school, in whom vaccine coverage was only 23% (median age at vaccination of 13 years, IQR 12–14), there was no significant vaccine effect (overall vaccine effectiveness 16%, 95% CI –34 to 47). In the same age range, in women who were HIV-negative, in whom vaccine coverage was 52% (median age at vaccination of 13 years, IQR 12–15), there was a significant reduction in HPV6, 11, 16, and 18 prevalence from 10% (95% CI 8 to 12) in the baseline survey to 5% (4 to 6) in the repeat survey, with an overall vaccine effectiveness of 55% (95% CI 36 to 69; table 3). Among women who were HIV-positive, in whom vaccine coverage was only 31% (median age at vaccination of 14 years, IQR 12–15), there was no significant vaccine effect (overall vaccine effectiveness 24%, 95% CI –62 to 64). Of note, however, no HPV6, 11, 16, or 18 infections were detected among the few (n=27) vaccinated HIV-positive women.

**Table 3:** Vaccination coverage in the repeat survey, human papillomavirus prevalence, and overall vaccine effectiveness for HPV types 6, 11, 16, and 18 by age, level of education, and HIV status

	**Baseline survey**			**Repeat survey**				Adjusted vaccine effectiveness (95% CI)*
	Number assessed	HPV-positive	HPV prevalence (95% CI)†	Vaccination coverage	Number assessed	HPV-positive	HPV prevalence (95% CI)†	
**Age 17–23 years**								
<6 years of school	479	52	11% (8 to 14)	23%	358	29	8% (6 to 11)	16% (−34 to 47)
≥6 years of school	613	82	13% (11 to 16)	63%	812	33	4% (3 to 6)	68% (51 to 79)
HIV-negative	754	75	10% (8 to 12)	52%	1072	52	5% (4 to 6)	55% (36 to 69)
HIV-positive	115	19	17% (10 to 25)	31%	93	10	11% (5 to 19)	24% (−62 to 64)
**Age 24–29 years**								
<6 years of school	167	20	12% (8 to 18)	1%	161	10	6% (3 to 11)	54% (−3 to 79)
≥6 years of school	242	19	8% (5 to 12)	14%	274	16	6% (3 to 9)	25% (−45 to 61)
HIV-negative	272	25	9% (6 to 13)	11%	341	12	4% (2 to 6)	55% (12 to 78)
HIV-positive	66	9	14% (6 to 24)	2%	97	13	13% (7 to 22)	−17% (−172 to 50)

Data are n or % unless otherwise stated. HPV=human papillomavirus.

* Overall vaccine effectiveness adjusted for level of education, HIV status, and lifetime number of sexual partners.

† Crude prevalence.

Vaccine coverage also varied among women aged 24–29 years by years of school completed (14% for ≥6 years *vs* 1% for <6 years) and HIV status (11% for HIV-negative *vs* 2% for HIV-positive), but vaccine coverage in this age group was insufficient to justify comparison of vaccine effectiveness by these strata. No HPV6, 11, 16, or 18 infections were detected among the few (n=41) vaccinated women aged 24–29 years.

Based on the overall vaccine effectiveness against HPV16 and 18, and against HPV31, 33, and 45 estimated among women aged 17–23 years, the lifetime risk of cervical cancer decreased from 2663 cases (uncertainty interval [UI] 1989–3462) per 100 000 women in the baseline survey (without vaccination) to 1660 cases (1239–2161) per 100 000 women in the repeat survey (with vaccination), and the annual age-standardised incidence rate decreased from 28·0 cases (UI 20·9–36·4) per 100 000 women in the baseline survey (without vaccination) to 17·5 cases (13·0–22·7) per 100 000 women in the repeat survey (with vaccination; table 4).

**Table 4:** Vaccine effectiveness against HPV16 and 18, and HPV31, 33, and 45, in women aged 17–23 years, with corresponding expected lifetime risk and annual age-standardised incidence rate of cervical cancer with and without vaccination in Rwanda

	**Vaccine effectiveness (95% CI)**			Lifetime risk (95% UI)*		Annual age-standardised incidence rate (95% UI)*	
	Overall	Total	Indirect	Without vaccination	With vaccination	Without vaccination	With vaccination
HPV16 and 18	48% (27 to 64)	66% (42 to 81)	32% (−1 to 54)	..	..	..	..
HPV31, 33, and 45	40% (15 to 58)	37% (1 to 60)	41% (8 to 62)	..	..	..	..
Cervical cancer	..	..	..	2663 (1989–3462)	1660 (1239–2161)	28·0 (20·9–36·4)	17·5 (13·0–22·7)

Age-standardised incidence rates were standardised on the world population. HPV=human papillomavirus. UI=uncertainty interval.

* Cases per 100 000 women, estimated among women in both the baseline survey (without vaccination) and repeat survey (with vaccination).

## Discussion

These cervical-cell-based impact-monitoring surveys in Rwanda showed a substantial overall effectiveness of the HPV vaccine (about 52%) against vaccine-targeted HPV types (HPV6, 11, 16, and 18) at a population level among women eligible for vaccination in the catch-up programme. Indirect effectiveness (herd immunity) contributed substantially to overall effectiveness. Total effectiveness of HPV vaccination against vaccine-targeted types (ie, real-world vaccine efficacy) was somewhat lower than the efficacy estimated in clinical trials,^11^ which was likely to be because the mean age at vaccination in these catch-up cohorts was 14 years (IQR 13–15), and some women in Rwanda would have already been sexually active when vaccinated.

The overall effectiveness against HPV vaccine types estimated in our survey among women offered the vaccine in the vaccination programme, who had vaccine coverage similar to that reported by Sayinzoga and colleagues,^3^ was lower than that recorded in repeat cross-sectional studies in Bhutan (ie, 93% among women younger than 27 years).^12^ In Bhutan, the estimated coverage of catch-up vaccination was higher than in Rwanda, at approximately 90%, and sexual behaviours were possibly different, as suggested by data collected in our surveys, which show a younger age at sexual debut in Rwanda (IQR 16–19 years) than in Bhutan (IQR 18–21 years).^12^ Overall effectiveness against HPV vaccine types can also be compared with reports of 87% in women aged 18–24 years in Malaysia (in whom HPV vaccine coverage was 75%), the first report from a upper-middle-income setting,^13^ as well as with 83% in girls aged 13–19 years in a meta-analysis of evidence available from 14 high-income countries.^14^

As in other similar studies,^4^ we detected a significant decrease in the prevalence of other alpha-9 HPV types (HPV31, 33, 35, 52, and 58), suggesting cross-protection of the four-valent vaccine. Because we did not detect any significant variation in HPV prevalence for any other type, observed prevalence reductions were unlikely to be confounded by changes in the sexual behaviour of the local population. Indeed, we could not identify any relevant variations in sexual behaviour reported by participants between the baseline and repeat surveys. We considered HPV prevalence estimates in the baseline and repeat surveys to be well comparable, as the same procedures were adopted for study recruitment, participant interviews, and testing, and our estimates were similarly adjusted for relevant participant characteristics.

Of note, self-reported vaccine coverage and overall vaccine effectiveness against vaccine-targeted HPV types were highly heterogeneous across subgroups of participants who were younger than 24 years, while vaccine coverage was low and overall vaccine effectiveness was non-significant in women aged 24–29 years. Overall effectiveness was higher among women who had completed more years of school and similar to that found in urine-based surveys done in schools from the same area of Kigali (78%, 95% CI 51–90).^4^ Vaccine coverage in the repeat survey in Rwanda was suboptimal, as could be expected from data reported by the national HPV vaccination programme.^3^ Predominantly school-based catch-up programmes do not adequately reach girls who are not attending school. This repeated observation is relevant to the design of a cervical cancer screening programme targeting catch-up vaccinated birth cohorts in Rwanda and catch-up campaigns in other countries that are newly introducing HPV vaccination. HIV-positive women had lower vaccine coverage than HIV-negative women, and showed non-significant vaccine effectiveness. We believe this finding to be confounded by shorter school attendance or higher probability of HPV infection before vaccination than in HIV-negative women. Indeed, although the overall reduction in lifetime incidence of cervical cancer among vaccinated birth cohorts is expected to be about 60%, a substantial disparity in the risk of cervical cancer should be expected among different subgroups of women within the same vaccinated birth cohorts.

Our study has some limitations. Participants were recruited in the catchment area of health centres in one district of Kigali, the capital of Rwanda, and so the study population might not be fully representative of the general population of the country, which is still largely rural. Furthermore, participation rates could not be estimated due to the uncertainty regarding the number of women who had been reached by the information campaign, and because exhaustive lists of the population residing in the catchment areas of health centres are not available. Also, women self-reported their vaccination status, and there might have been some misreporting. To assess the sensitivity of our total and indirect vaccine effectiveness estimates to the uncertainty about reported vaccination status (note that overall effectiveness estimates are not affected by the reported vaccination status or its misclassification), we recalculated total and indirect vaccine effectiveness, first excluding from the analysis all women who were unsure about their vaccination status, and then considering these women as vaccinated. No substantial effect due to possible misclassification was observed. Finally, although it is possible that heterogeneity of vaccine effectiveness across Rwanda might not be entirely captured in our estimate, no other data are yet available; therefore, we have drawn our projections of lifetime cervical cancer risk under the provisional assumption that the overall adjusted vaccine effectiveness estimated from our surveys was representative of the whole country.

In conclusion, this study builds upon our previous observation of a strong impact of HPV vaccination among school girls in Kigali^4^ and expands impact assessment to a population sample of older sexually active women. It also highlights differences in vaccine coverage and impact at a population level according to school attendance and HIV status. Continued assessment will be necessary to monitor the long-term impact of HPV vaccination in Rwanda, especially among birth cohorts who had been vaccinated routinely with a higher coverage in schools at age 12 years. Our encouraging observations will hopefully reinforce the commitment of international donors to support HPV vaccination in low-income and middle-income countries. Similarly, these findings might motivate public health authorities in Rwanda to ensure the sustainability of the HPV vaccination programme and also help the impetus to design cost-effective cervical cancer screening strategies adapted to the considerable decrease in prevalence of HPV16 and 18 in vaccinated populations.

## Data sharing

External researchers can make written requests to the International Agency for Research on Cancer for sharing of data and study protocol. Requests will be assessed on a case-by-case basis in consultation with the lead and co-investigators. All data sharing will abide by rules and policies defined by the involved parties. Data sharing mechanisms will ensure that the rights and privacy of individuals participating in research will be protected at all times. The model code used to project the estimated impact of HPV vaccination on cervical cancer burden in Rwanda is available at https://gitlab.com/iarc-miarc/methis/methis.atlas.

## Declaration of interests

DAMH is minority shareholder of Self-screen, a spin-off company of VU University Medical Center (currently known as Amsterdam UMC, location Vrije Universiteit Amsterdam); Self-screen develops, manufactures, and licences high-risk HPV and methylation marker assays for cervical cancer screening and holds patents on these tests. All other authors declare no competing interests.

## References

[1] Sung H, Ferlay J, Siegel RL (2021). Global cancer statistics 2020: GLOBOCAN estimates of incidence and mortality worldwide for 36 cancers in 185 countries. CA Cancer J Clin.

[2] Binagwaho A, Wagner CM, Gatera M, Karema C, Nutt CT, Ngabo F (2012). Achieving high coverage in Rwanda's national human papillomavirus vaccination programme. Bull World Health Organ.

[3] Sayinzoga F, Umulisa MC, Sibomana H, Tenet V, Baussano I, Clifford GM (2020). Human papillomavirus vaccine coverage in Rwanda: a population-level analysis by birth cohort. Vaccine.

[4] Baussano I, Sayinzoga F, Tshomo U (2021). Impact of human papillomavirus vaccination, Rwanda and Bhutan. Emerg Infect Dis.

[5] Ngabo F, Franceschi S, Baussano I (2016). Human papillomavirus infection in Rwanda at the moment of implementation of a national HPV vaccination programme. BMC Infect Dis.

[6] Franceschi S, Clifford GM, Baussano I (2018). Options for design of real-world impact studies of single-dose vaccine schedules. Vaccine.

[7] de Roda Husman AM, Snijders PJ, Stel HV, van den Brule AJ, Meijer CJ, Walboomers JM (1995). Processing of long-stored archival cervical smears for human papillomavirus detection by the polymerase chain reaction. Br J Cancer.

[8] Jacobs MV, Walboomers JM, Snijders PJ (2000). Distribution of 37 mucosotropic HPV types in women with cytologically normal cervical smears: the age-related patterns for high-risk and low-risk types. Int J Cancer.

[9] Halloran ME, Struchiner CJ, Longini IM (1997). Study designs for evaluating different efficacy and effectiveness aspects of vaccines. Am J Epidemiol.

[10] Bonjour M, Charvat H, Franco EL (2021). Global estimates of expected and preventable cervical cancers among girls born between 2005 and 2014: a birth cohort analysis. Lancet Public Health.

[11] Arbyn M, Xu L, Simoens C, Martin-Hirsch PP (2018). Prophylactic vaccination against human papillomaviruses to prevent cervical cancer and its precursors. Cochrane Database Syst Rev.

[12] Baussano I, Tshomo U, Tenet V (2020). Prevalence of human papillomavirus and estimation of human papillomavirus vaccine effectiveness in Thimphu, Bhutan, in 2011–2012 and 2018: a cross-sectional study. Ann Intern Med.

[13] Khoo SP, Muhammad Ridzuan Tan NA, Rajasuriar R (2022). Changes in genital human papillomavirus (HPV) prevalence among urban females a decade after the Malaysian HPV vaccination program. PLoS One.

[14] Drolet M, Bénard É, Pérez N (2019). Population-level impact and herd effects following the introduction of human papillomavirus vaccination programmes: updated systematic review and meta-analysis. Lancet.

