# Supplementary material for: Human papillomavirus vaccine effect against human papillomavirus infection in Rwanda: evidence from repeated cross-sectional cervical-cell-based surveys
Source: Lancet Glob Health. 2023 May 16;11(7):e1096–104. doi: 10.1016/S2214-109X(23)00193-6 (PMC10282073; doi:10.1016/S2214-109X(23)00193-6)
Supplement: Supplementary appendix [file mmc1.pdf]

THE LANCET
Global Health

Supplementary appendix

This appendix formed part of the original submission and has been peer reviewed. We post it as supplied by the authors.

Supplement to: Sayinzoga F, Tenet V, Heideman DAM, et al. Human papillomavirus vaccine effect against human papillomavirus infection in Rwanda: evidence from repeated cross-sectional cervical-cell-based surveys. *Lancet Glob Health* 2023; published online May 16. https://doi.org/10.1016/S2214-109X(23)00193-6.

Appendix

Table of Contents

Methods S1: Methods adopted to assess the lifetime risk of cervical cancer. 2
Table S1: List of health-centres in Nyarugenge District 5
Table S2: All ages and age-specific prevalence ratios and vaccine effectiveness in Rwanda with corresponding 95% CI for positivity for other alpha-7 and other alpha-9 HPV types 6
Table S3: All ages and age-specific HPV prevalence, prevalence ratios, and vaccine effectiveness for vaccine-targeted (HPV6, 11, 16, and 18) and other alpha-9 HPV types - Sensitivity 1: "Not sure" if vaccinated are excluded 7
Table S4: All ages and age-specific HPV prevalence, prevalence ratios, and vaccine effectiveness for vaccine-targeted (HPV6, 11, 16, and 18) and other alpha-9 HPV types - Sensitivity 2: "Not sure" if vaccinated are taken into account with the "vaccinated" 8
Table S5: All ages and age-specific HPV prevalence, prevalence ratios, and vaccine effectiveness for vaccine-targeted (HPV6, 11, 16, and 18) HPV types. Sensitivity analysis restricted to the 9 health-centres participating in both surveys, with health-centres modelled as a clustering variable. 9
Figure S1: Analytical framework used to assess the impact of HPV vaccination in Rwanda. 10
Figure S2: Study flow diagram Rwanda baseline (2013–2014) and repeat (2019–2020) surveys 11
Annex 1: Informed Consent Form 12
Annex 2: Risk Factor Questionnaire 14

Methods S1: Methods adopted to assess the lifetime risk of cervical cancer.

Derivation of the formula for estimating the contribution to the cumulative incidence of cervical cancer for a particular age group taking account of the competing risk of dying from any cause before being diagnosed with cervical cancer.

For a given cohort of women, the expected cumulative number of cervical cancers between age 15 and 79 years in the absence of vaccination was calculated by using 5-year age group-specific cervical cancer incidence rate projections from the Global Cancer Observatory (GLOBOCAN) (http://gco.iar.fr) project[1,2] and 5-year age group-specific mortality rates were obtained from United Nations, Department of Economic and Social Affairs, Population Division.[3]

Let A_0 be the age group 15-19 years, …, A_{12} be the age group 75-79 years. Let us denote by λ_C and λ_M respectively cervical cancer incidence rate and mortality rate, both assumed to be piecewise constant functions on each five-year age group. That is, for $i = 0, \dots, 12$, $\lambda_{C,i}$ and $\lambda_{M,i}$ denote respectively the constant A_i-specific cervical cancer incidence and mortality rates. Let us also designate by A_i^L and A_i^U respectively the lower and upper bound of age interval A_i, with for $i > 0$, $A_i^L = A_{i-1}^U$. Then, the survival free from event (i.e., cervical cancer diagnosis or death) by age A_I^U for a cohort of women aged 15 at the start of follow-up can be calculated according to the formula:

$$S(A_I^U) = S(A_{I+1}^L) = \exp\left(-\int_{A_0^L}^{A_I^U} \big(\lambda_C(u) + \lambda_M(u)\big)du\right)$$

$$= \exp\left(-5\sum_{i=0}^{I}\big(\lambda_{C,i} + \lambda_{M,i}\big)\right)$$

Now, the cumulative incidence of cervical cancer by age A_I^U can be calculated as:

$$CumI(A_I^U) = \int_{A_0^L}^{A_I^U} \lambda_C(u)S(u)du$$

$$= \sum_{i=0}^{I} \lambda_{C,i}\int_{A_i^L}^{A_i^U} S(u)du$$

$$= \sum_{i=0}^{I} CumI_i$$

We then have:

$$CumI_i = \lambda_{C,i}\int_{A_i^L}^{A_i^U} S(u)du$$

$$= \lambda_{C,i}\int_{A_i^L}^{A_i^U} \exp\left(-\int_{A_0^L}^{u}\big(\lambda_C(v) + \lambda_M(v)\big)dv\right)du$$

$$= \lambda_{C,i}\int_{A_i^L}^{A_i^U} \exp\left(-\int_{A_0^L}^{A_i^L}\big(\lambda_C(v) + \lambda_M(v)\big)dv\right)\exp\left(-\int_{A_i^L}^{u}\big(\lambda_C(v) + \lambda_M(v)\big)dv\right)du$$

$$= \lambda_{C,i}\exp\left(-5\sum_{k=0}^{i-1}\big(\lambda_{C,k} + \lambda_{M,k}\big)\right)\int_{A_i^L}^{A_i^U} \exp\left(-\int_{A_i^L}^{u}\big(\lambda_C(v) + \lambda_M(v)\big)dv\right)du$$

$$= -\frac{\lambda_{C,i}}{\lambda_{C,i} + \lambda_{M,i}} \exp\left(-5\sum_{k=0}^{i-1}(\lambda_{C,k} + \lambda_{M,k})\right)\left(\exp\left(-5(\lambda_{C,i} + \lambda_{M,i})\right) - 1\right)$$

$$= -\frac{\lambda_{C,i}}{\lambda_{C,i} + \lambda_{M,i}}\left(S(A_i^U) - S(A_{i-1}^U)\right)$$

$$= \frac{\lambda_{C,i}}{\lambda_{C,i} + \lambda_{M,i}}\left(S(A_i^L) - S(A_i^U)\right)$$

Based on the assumption that the age group-specific incidence rates were stable across birth cohorts, the cumulative incidence between ages 15 and 79 was then obtained by summing the age group-specific contributions:$\mathrm{CumI}_{15-79} = \sum_{i=0}^{12} \mathrm{CumI}_i$. Finally, the cumulative number of cervical cancers was calculated by multiplying this cumulative incidence by the size of the cohort at age 15 years, also estimated from the GLOBOCAN data. Uncertainty intervals were estimated with Monte-Carlo simulation combining uncertainty about cervical cancer incidence as reported in GLOBOCAN 2020 and HPV distribution in cervical cancers as reported by Guan et al.[4] Our estimates were obtained based on Monte Carlo simulation with 100 repetitions as this number of repetitions provides stable estimates. Of note, no burn-in is needed for the applied Monte Carlo simulation since this used to assess the uncertainty of our estimates rather than fitting our model to target statistics.

Of note, we recently validated GLOBOCAN estimates for Rwanda[5] using an independent method to quantify the cervical cancer incidence based on age-specific population-based HPV prevalence. The independent estimates are consistent with those reported in GLOBOCAN.

Finally, the procedures adopted to estimate the expected lifetime risk (LTR) of cervical cancer and the annual age-standardised incidence rate, are documented at the following website:
https://iarc-miarc.gitlab.io/methis/methis.atlas/reference/atlas_ui.html#references

References

1. Sung H, Ferlay J, Siegel RL, et al. Global Cancer Statistics 2020: GLOBOCAN Estimates of Incidence and Mortality Worldwide for 36 Cancers in 185 Countries. *CA Cancer J Clin* 2021; **71**(3): 209–49.

2. Ferlay J, Colombet M, Soerjomataram I, et al. Estimating the global cancer incidence and mortality in 2018: GLOBOCAN sources and methods. *Int J Cancer* 2019; **144**(8): 1941–53.

3. United Nations Department of Economic and Social Affairs Population Division. World Population Prospects 2019.

4. Guan P, Howell-Jones R, Li N, et al. Human papillomavirus types in 115,789 HPV-positive women: A meta-analysis from cervical infection to cancer. *Int J Cancer* 2012; **131**(10): 2349–59.

5. Schulte-Frohlinde R, Georges D, Clifford GM, Baussano I. Predicting Cohort-Specific Cervical Cancer Incidence From Population-Based Surveys of Human Papilloma Virus Prevalence: A Worldwide Study. *American journal of epidemiology* 2022; **191**(3): 402–12.

Table S1: List of health-centres in Nyarugenge District

HEALTH CENTRES:
1 = MUHIMA HOSPITAL<br>2 = MUHIMA HEALTH CENTER<br>3 = BUTAMWA HEALTH CENTER<br>4 = RUGARAMA HEALTH CENTER<br>5 = MWENDO HEALTH CENTER<br>6 = CORNUM HEALTH CENTER<br>7 = GITEGA HEALTH CENTER<br>8 = KABUSUNZU HEALTH CENTER<br>9 = BIRYOGO HEALTH CENTER<br>10 = KANYINYA HEALTH CENTER<br>11 = NYARURENZI HEALTH CENTER

Table S2: All ages and age-specific prevalence ratios and vaccine effectiveness in Rwanda with corresponding 95% CI for positivity for other alpha-7 and other alpha-9 HPV types

HPV type*	Age-group, years	Type of effectiveness	Baseline survey		Repeat survey		Adjusted VE % (95% CI)†
			N	HPV+ (%)	N	HPV+ (%)	
**Other alpha-7**	17–29	Overall‡	1501	107 (7%)	1639	99 (6%)	17% (-10–38)
		Indirect§	1478	104 (7%)	979	54 (6%)	24% (-7–45)
		Total‖	1478	104 (7%)	638	45 (7%)	14% (-27–42)
	17–23	Overall‡	1092	86 (8%)	1191	78 (7%)	23% (-6–45)
		Indirect§	1072	83 (8%)	585	34 (6%)	34% (-2–56)
		Total‖	1072	83 (8%)	597	44 (7%)	16% (-26–44)
	24–29	Overall‡	409	21 (5%)	448	21 (5%)	-1% (-84–47)
		Indirect§	406	21 (5%)	394	20 (5%)	-2% (-93–46)
		Total‖	406	21 (5%)	41	1 (2%)	14% (-565–89)
**Non-alpha 7/9**	17–29	Overall‡	1501	403 (27%)	1639	427 (26%)	8% (-4–19)
		Indirect§	1478	394 (27%)	979	245 (25%)	8% (-6–20)
		Total‖	1478	394 (27%)	638	181 (28%)	7% (-10–21)
	17–23	Overall‡	1092	318 (29%)	1191	341 (29%)	7% (-7–19)
		Indirect§	1072	310 (29%)	585	168 (29%)	8% (-9–22)
		Total‖	1072	310 (29%)	597	173 (29%)	6% (-12–21)
	24–29	Overall‡	409	85 (21%)	448	86 (19%)	9% (-20–31)
		Indirect§	406	84 (21%)	394	77 (20%)	10% (-20–32)
		Total‖	406	84 (21%)	41	8 (20%)	6% (-89–54)

*Other alpha-7 types (HPV39, 45, 59, and 68); non-alpha 7/9 types (HPV26, 30, 32, 34, 40, 42, 43, 44, 51, 53, 54, 55, 56, 64, 66, 67, 69, 70, 72, 73, 81, 82, 83, 84, 85, 86, 89, and 90). †Adjusted for age, education, HIV status, and lifetime number of sexual partners. ‡Entire baseline group compared to entire repeat group. §Unvaccinated baseline group compared to entire repeat group. ‖Unvaccinated baseline group compared to vaccinated repeat group. HPV=human papillomavirus. VE=vaccine effectiveness.

Table S3: All ages and age-specific HPV prevalence, prevalence ratios, and vaccine effectiveness for vaccine-targeted (HPV6, 11, 16, and 18) and other alpha-9 HPV types Sensitivity 1: "Not sure" if vaccinated are excluded

HPV type	Age-group, years	Type of effectiveness	Baseline survey		Repeat survey		Adjusted VE % (95% CI)†
			N	HPV+ (%)	N	HPV+ (%)	
**HPV6, 11, 16, and 18**	All ages	Overall‡	1501	173 (12%)	1639	89 (5%)	47% (31–60)
		Indirect§	1478	169 (11%)	871	57 (7%)	36% (13–53)
		Total‖	1478	169 (11%)	638	20 (3%)	70% (52–82)
	17–23	Overall‡	1092	134 (12%)	1191	63 (5%)	52% (35–65)
		Indirect§	1072	130 (12%)	491	33 (7%)	40% (12–60)
		Total‖	1072	130 (12%)	597	20 (3%)	69% (50–81)
	24–29	Overall‡	409	39 (10%)	448	26 (6%)	37% (-5–62)
		Indirect§	406	39 (10%)	380	24 (6%)	34% (-11–61)
		Total‖	406	39 (10%)	41	0 (0%)	100% (NA)

†Adjusted for age, education, HIV status, and lifetime number of sexual partners. ‡Entire baseline group compared to entire repeat group. §Unvaccinated baseline group compared to entire repeat group. ‖Unvaccinated baseline group compared to vaccinated repeat group. HPV=human papillomavirus. VE=vaccine effectiveness.

Table S4: All ages and age-specific HPV prevalence, prevalence ratios, and vaccine effectiveness for vaccine-targeted (HPV6, 11, 16, and 18) and other alpha-9 HPV types - Sensitivity 2: "Not sure" if vaccinated are taken into account with the "vaccinated"

HPV type	Age-group, years	Type of effectiveness	Baseline survey		Repeat survey		Adjusted VE % (95% CI)†
			N	HPV+ (%)	N	HPV+ (%)	
**HPV6, 11, 16, and 18**	All ages	Overall‡	1501	173 (12%)	1639	89 (5%)	47% (31–61)
		Indirect§	1478	169 (11%)	871	57 (7%)	36% (13–53)
		Total‖	1478	169 (11%)	746	32 (4%)	60% (40-73)
	17–23	Overall‡	1092	134 (12%)	1191	63 (5%)	52% (35–65)
		Indirect§	1072	130 (12%)	491	33 (7%)	40% (12–60)
		Total‖	1072	130 (12%)	691	30 (4%)	61% (40–74)
	24–29	Overall‡	409	39 (10%)	448	26 (6%)	37% (-5–62)
		Indirect§	406	39 (10%)	380	24 (6%)	34% (-11–61)
		Total‖	406	39 (10%)	55	2 (4%)	51% (-104–88)

†Adjusted for age, education, HIV status, and lifetime number of sexual partners. ‡Entire baseline group compared to entire repeat group. §Unvaccinated baseline group compared to entire repeat group. ‖Unvaccinated baseline group compared to vaccinated repeat group. HPV=human papillomavirus. VE=vaccine effectiveness.

Table S5: All ages and age-specific HPV prevalence, prevalence ratios, and vaccine effectiveness for vaccine-targeted (HPV6, 11, 16, and 18) HPV types. Sensitivity analysis restricted to the 9 health-centres participating in both surveys, with health-centres modelled as a clustering variable.

HPV type	Age-group, years	Type of effectiveness	Baseline survey		Repeat survey		Adjusted VE % (95% CI)†	ICC
			N	HPV+ (%)	N	HPV+ (%)		
**HPV6, 11, 16, and 18**	All ages	Overall‡	1501	173 (12%)	1345	78 (6%)	45% (22–61)	0·021 (0·004–0·093)
		Indirect§	1478	169 (11%)	810	61 (8%)	29% (-7–52)	0·028 (0·006–0·122)
		Total‖	1478	169 (11%)	513	17 (3%)	69% (54–79)	0·018 (0·003–0·096)
	17–23	Overall‡	1092	134 (12%)	984	55 (6%)	50% (30–64)	0·025 (0·005–0·122)
		Indirect§	1072	130 (12%)	185	38 (8%)	32% (0–54)	0·029 (0·005–0·142)
		Total‖	1072	130 (12%)	490	17 (4%)	68% (51–80)	0·027 (0·005–0·139)
	24–29	Overall‡	409	39 (10%)	361	23 (6%)	33% (-28–64)	0·000 (0·000–1·000)
		Indirect§	406	39 (10%)	325	23 (7%)	27% (-39–61)	0·000 (0·000–1·000)
		Total‖	406	39 (10%)	23	0 (0%)	100% (NA)	0·000 (0·000–1·000)

†Adjusted for age, education, HIV status, and lifetime number of sexual partners. ‡Entire baseline group compared to entire repeat group. §Unvaccinated baseline group compared to entire repeat group. ‖Unvaccinated baseline group compared to vaccinated repeat group. HPV=human papillomavirus. VE=vaccine effectiveness. ICC=Intraclass Correlation Coefficient.

Figure S1: Analytical framework used to assess the impact of HPV vaccination in Rwanda.

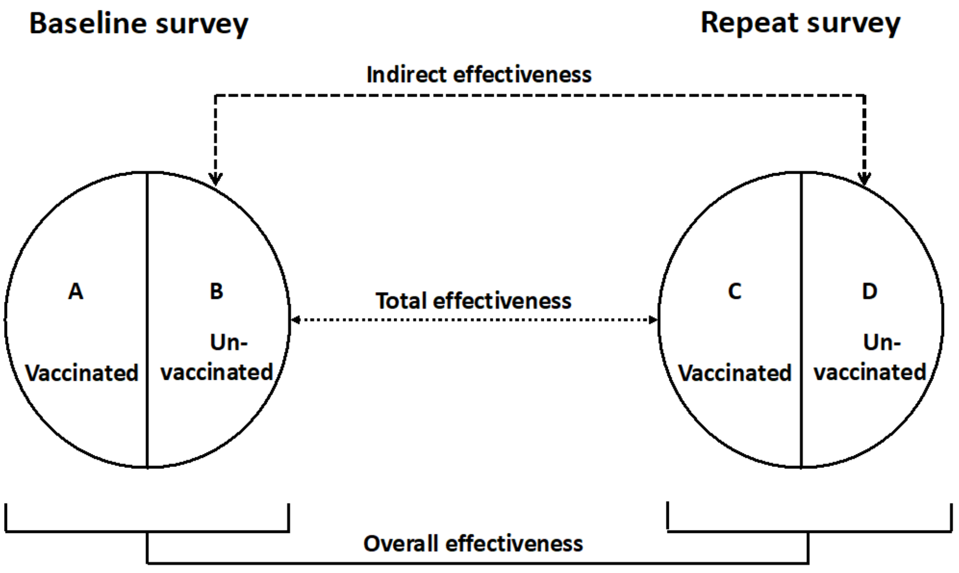

Vaccine effectiveness was calculated as VE = (1-PR)%, where PR is a prevalence ratio (PR). Each VE type compares different groups of women based upon reported vaccination status.

1) Overall effectiveness estimates HPV prevalence reduction over time attributable to vaccination irrespective of the reported vaccination status of each individual. It was computed by comparing type-specific HPV prevalence in all women (unvaccinated and vaccinated), recruited in the baseline and repeat surveys.

Pr(C+D) / Pr(A+B) = Overall PR

2) Total effectiveness estimates vaccine efficacy (i.e. similar to measures made in clinical trials) from real-life settings. It was computed by comparing the type-specific HPV prevalence in unvaccinated women in the baseline survey and vaccinated women in the repeat survey.

Pr(C) / Pr(B) = Total PR

3) Indirect effectiveness estimates the impact of HPV vaccination among unvaccinated individuals in a vaccinated population. It was computed by comparing the type-specific HPV prevalence in unvaccinated women in the baseline and repeat surveys.

Pr(D) / Pr(B) = Indirect PR

Where Pr(•) is the type-specific HPV prevalence in each participant group.

HPV=human papillomavirus.

Figure S2: Study flow diagram Rwanda baseline (2013–2014) and repeat (2019–2020) surveys

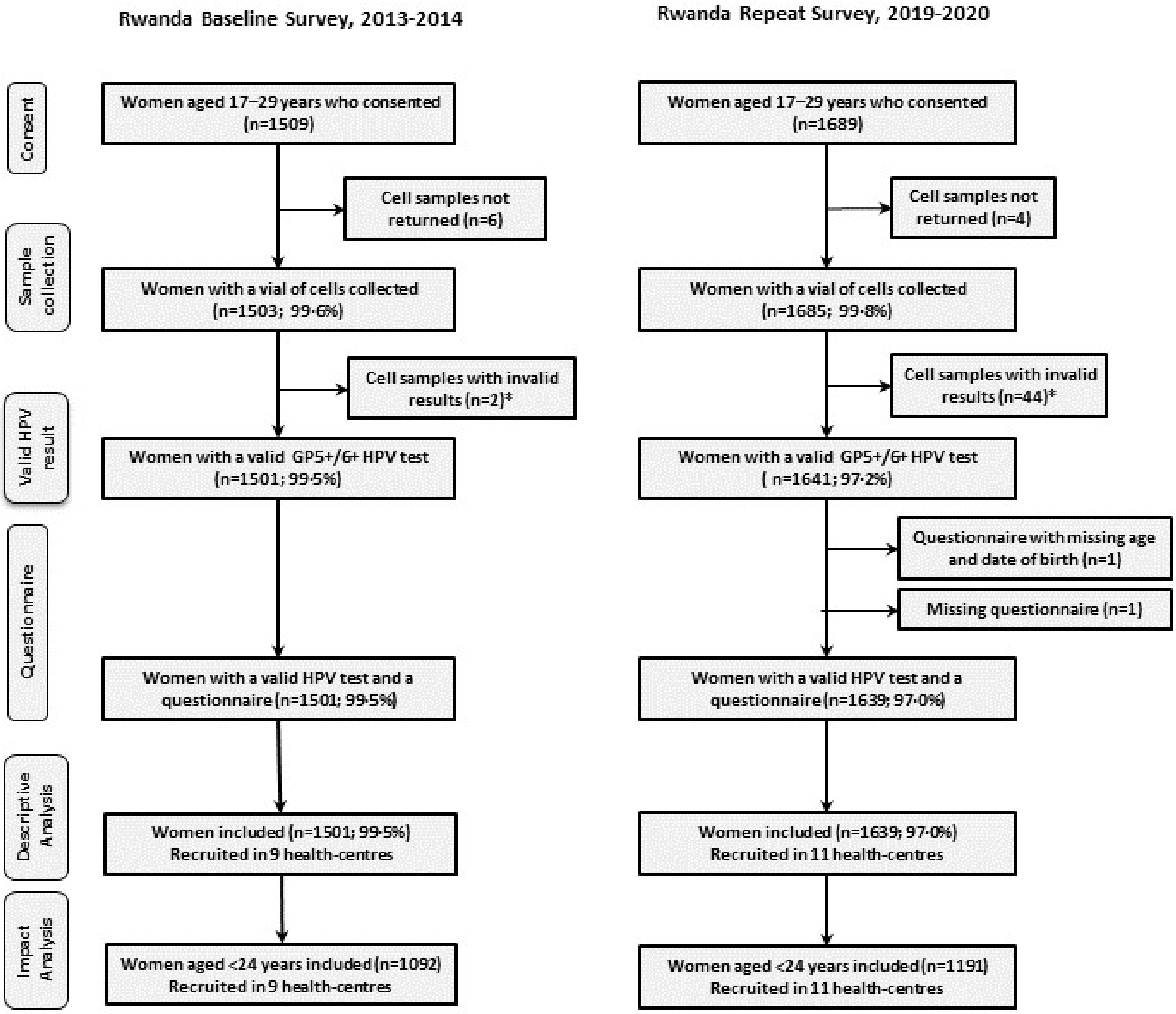

*Invalid results means the HPV assay GP5+/6+ results were negative for β -globin and HPV results could not be determined.
HPV= human papillomavirus.

Note that, all mentally and physically competent women were eligible for the study, regardless of their marital status, with exception of those who were known to be pregnant. All participants were residents of Nyarugenge District, Kigali and were recruited in two ways: 1) A population-based invitation of women aged 18–69 years residing in pre-defined areas of Nyarugenge District was made through community workers and community meetings. Women were invited to attend the Reproductive Health Department of the district hospital (Muhima). Participation rates could not be estimated due to the uncertainty on the number of women who had been reached by the information campaign and because exhaustive lists of the population resident in the catchment areas of health centres are not available. 2) An additional opportunistic approach was used to recruit women aged ≤29 years who were spontaneously attending Muhima hospital or health centers in the Nyarugenge District. Principal reasons for consultation were family planning, childhood vaccination, post-natal care services and HIV-related services. Study procedures were undertaken in the centre where the woman was consulting and very few refusals were recorded. Following signature of an informed consent form, a structured risk questionnaire was administered to all study participants, and data transferred electronically to IARC via a specifically designed on-line webpage/application."

Annex 1: Informed Consent Form

HUMAN PAPILLOMAVIRUS (HPV) PREVALENCE SURVEY
INFORMED CONSENT FORM

The Ministry of Health in Rwanda is working with the International Agency for Research on Cancer to conduct a study on the prevalence of human papillomavirus (a virus that is commonly found in the genital tract) in Rwanda. It will help to assess the impact of the HPV vaccination program to prevent cervical cancer in the future. You are being invited to participate in this study, which has been approved by the Rwandan National Ethical Committee (contacts: Dr. ……………………… at ………….).

STUDY PROCEDURES

A trained health worker will place a speculum in your vagina and take a specimen of cells from the surface of your cervix using a soft brush. In the event that any gynecological abnormalities are seen during the sample taking, you will be referred for appropriate medical evaluation and treatment.

You will be asked questions about your background, education, pregnancies, family planning, health, smoking and lifestyle. Some of the questions will be personal. You can refuse to answer any question that bothers you.

TEST AND TEST RESULTS

The specimen of cells from your cervix will be used to detect human papillomavirus and other factors potentially associated with cervical cancer. The tests will be purely research tests, with the aim to improve cervical cancer prevention in the future.

BENEFITS AND RISKS

The HPV test is not informative for your clinical care as you are less than 30 years, and will be used for research purposes only.

The examination to collect these specimens is a routine procedure and considered safe. No complications or discomfort is expected except for the usual discomfort that you may feel from a routine gynecological exam.

CONFIDENTIALITY

All the information you provide and the results from your biological specimens will be kept in strict confidence. Your name will not be associated with the results of this study. Information from the interview and your specimens will be identified by a study number only. Data collected will only be used to develop statistical summaries to be presented in published reports.

PARTICIPATION

If you decide to participate in this study you may withdraw your consent at any time afterwards. If at any time you have questions about the study you may contact Dr ……………., Head of Child and Maternal Health, Rwandan

Ministry of Health at …………………... If you have any question related to your rights as a participant contact the chairperson of the Rwanda National Ethics Committee Dr …………………. at ………………….. or secretary of the Rwanda National Ethics Committee Dr ……………………………. at ……………………………….

CONSENT AGREEMENT

By signing below, you indicate that you have read this document and that you understand its contents. Your signature below means that you freely agree to participate in this study. A duplicate of the signed consent form will be given to you.

______________	___________________________	______________________
DATE	PARTICIPANT'S NAME	SIGNATURE
______________	___________________________	______________________
DATE	NAME OF STUDY STAFF	SIGNATURE

Annex 2: Risk Factor Questionnaire

International Agency for Research on Cancer

World Health Organization

::: IARC HUMAN PAPILLOMAVIRUS (HPV) PREVALENCE SURVEY :::

IARC HUMAN PAPILLOMAVIRUS (HPV) PREVALENCE SURVEY

RISK FACTOR QUESTIONNAIRE

FILL IN INSTRUCTIONS

- Preferably use a pencil
- If you use a pen and data was noted by error, please cross out the wrong answer with a single line to leave it visible, write the correct answer in the margins:
  ~~| 8 | 1 |~~ 3 1
- Write numbers clearly and as large as possible in the box
- For dates, always use : DD MM YYYY
- For numeric values, note one number per box and align right: | 0 | 4 | 0 |
- Check the box with a cross mark: ☒
- If a data is not available, not known by the interviewer, or does not apply, please fill all the boxes with number "9", e.g.: | 9 | / | 9 | 9 | / | 9 | 9 | 9 |
- For ages, try to get at least an approximation
- Do not leave any box blank.

ADMINISTRATIVE DATA

STUDY ID NUMBER: |_1_|_7_|_1_| - |___|___|___|___|

STUDY ID LABEL

1. STUDY CENTRE:

- ☐ 1 = MUHIMA **HOSPITAL**
- ☐ 2 = MUHIMA **HEALTH CENTER**
- ☐ 3 = BUTAMWA HEALTH CENTER
- ☐ 4 = RUGARAMA HEALTH CENTER
- ☐ 5 = MWENDO HEALTH CENTER
- ☐ 6 = CORNUM HEALTH CENTER
- ☐ 7 = GITEGA HEALTH CENTER
- ☐ 8 = KABUSUNZU HEALTH CENTER
- ☐ 9 = BIRYOGO HEALTH CENTER
- ☐ 10 = KANYINYA HEALTH CENTER
- ☐ 11 = NYARURENZI HEALTH CENTER

2. DATE OF INTERVIEW: |___|___| - |___|___| - |___|___|___|___|

DAY MONTH YEAR

3. REASON FOR CONSULTING THIS HEALTH CENTER/HOSPITAL:

- ☐ 1 = ANTENATAL CARE
- ☐ 2 = FAMILY PLANNING
- ☐ 3 = VACCINATION
- ☐ 4 = HIV/ANTIRETROVIRAL SERVICES (ARV)
- ☐ 5 = MEDICAL CONSULTATION
- ☐ 6 =OTHER ______________________________

4. NAME OF INTERVIEWER: ____

5. DID THE WOMAN SIGN THE CONSENT FORM?

- ☐ 1 = NO [DO NOT COMPLETE REST OF QUESTIONNAIRE]
- ☐ 2 = YES

DEMOGRAPHIC DATA (1/2)

6. **WHAT IS YOUR DATE OF BIRTH**

|___|___| - |___|___| - |___|___|___|___|
DAY MONTH YEAR
(99 if unknown)

7. **HOW OLD ARE YOU?**

AGE |___|___|

8. **WHERE WERE YOU BORN?**

☐ 1 = KIGALI
☐ 2 = RWANDA, OUTSIDE KIGALI
☐ 3 = CONGO RDC
☐ 4 = UGANDA
☐ 5 = BURUNDI
☐ 6 = ABROAD, ANOTHER COUNTRY, PLEASE SPECIFY: ______________________________

9. **WHAT IS YOUR OCCUPATION?**

Try to find the nearest occupation in terms of income and educational requirements

☐ 1 = HOUSEWIFE
☐ 2 = SHOPKEEPER/SALESWOMAN
☐ 3 = MANUAL WORKER
☐ 4 = CLERICAL STAFF
☐ 5 = TEACHER/HEALTH WORKER
☐ 6 = FARMER
☐ 7 = STUDENT
☐ 8 = OTHER, PLEASE SPECIFY ______________________________

10. **WHAT IS YOUR CURRENT MARITAL STATUS?**

☐ 1 = NEVER MARRIED
☐ 2 = COHABITING OUTSIDE MARRIAGE
☐ 3 = MARRIED
☐ 4 = WIDOWED
☐ 5 = SEPARATED/DIVORCED

(for 2–5) HOW MANY TIMES HAVE YOU MARRIED (OR COHABITED)?
☐ 1
☐ 2
☐ 3
☐ 4+

11. **CAN YOU READ ANY LANGUAGE?**

☐ 1 = NO
☐ 2 = YES

DEMOGRAPHIC DATA (2/2)

12. **HAVE YOU BEEN TO SCHOOL?**

☐ 1 = NO → GO TO QUESTION 15

☐ 2 = YES] **HOW MANY YEARS OF EDUCATION HAVE YOU COMPLETED?** |____|____| YEARS
(99 if unknown)

13. **HAVE YOU BEEN IN PRIMARY SCHOOL GRADE 6 IN ANY YEAR SINCE 2011?**

☐ 1 = NO

☐ 2 = YES

14. **WERE YOU IN LOWER SECONDARY SCHOOL GRADE 3 IN 2012 OR 2013?**

☐ 1 = NO

☐ 2 = YES

HPV VACCINATION

15. **AS FAR AS YOU KNOW, HAVE YOU BEEN VACCINATED AGAINST HUMAN PAPILLOMAVIRUS?**

How to remember if you were given the vaccine and not make confusions with other vaccines/injections that you may have received over the years?

- *Vaccine was given to **girls only***
- *Vaccine was usually given in **primary school grade 6 since 2011***
- *Vaccine could also be given in **secondary school grade 3 in 2012/13***
- *Vaccine was given most often **at school** and marginally in health center*
- *Vaccine was usually given **in 3 doses** at 0, 2, and 6 months. But it could happen that only 1 or 2 doses only were given.*

☐ 1 = NO]
☐ 2 = NOT SURE] → GO TO QUESTION 18

☐ 3 = YES

16. **HOW OLD WERE YOU WHEN YOU RECEIVED THE HPV VACCINE?**

AGE |____|____|
(99 if unknown)

17. **NUMBER OF DOSES OF HPV VACCINE RECEIVED**

☐ 1

☐ 2

☐ 3

☐ 9 = CAN'T REMEMBER] **DO YOU REMEMBER IF YOU RECEIVED 1 OR MORE THAN 1 DOSE?**

☐ 1

☐ MORE THAN 1

☐ CAN'T REMEMBER

Reproductive History

18. **Have you ever been pregnant?** *(pregnancy = live birth, still birth, abortion, ...)*

☐ 1 = No → go to question 19

☐ 2 = Yes, **how many times have you been pregnant?** |____|____| pregnancy(ies)
(99 if unknown)

How old were you at your first pregnancy? Age |____|____|
(99 if unknown)

Sexual History (1/2)

19. **Throughout your life, how many different men you have had sexual intercourse with (including your current husband/cohabiting partner)?**

☐ 0 → go to question 26
☐ 1
☐ 2
☐ 3
☐ 4+
☐ Prefer not to answer

20. **How old were you when you first had sexual intercourse with a man?**

Age |____|____|
(99 if unknown)

21. **How old was your first partner when you first had sex with him?**

Age |____|____|
(99 if unknown)

22. **To your knowledge, did your current partner/husband ever have a sexual intercourse with another woman <u>before</u> you started having sexual intercourse with him?**

☐ 1 = No
☐ 2 = Probably yes
☐ 3 = Yes

23. **To your knowledge, did your current partner/husband ever have a sexual intercourse with another woman <u>after</u> you started having sexual intercourse with him?**

☐ 1 = No
☐ 2 = Probably yes
☐ 3 = Yes

SEXUAL HISTORY (2/2)

24. **HOW MANY DIFFERENT SEXUAL PARTNERS (INCLUDING YOUR HUSBAND/COHABITING PARTNER) DID YOU HAVE SEX WITH WITHIN THE LAST 12 MONTHS?**

☐ 0
☐ 1
☐ 2
☐ 3
☐ 4+
☐ PREFER NOT TO ANSWER

25. **HAVE YOU EVER RECEIVED ANY CASH/KIND FOR SEX IN YOUR LIFE?**

☐ 1 = NO
☐ 2 = YES
☐ 3 = PREFER NOT TO ANSWER

SMOKING HISTORY

26. **HAVE YOU EVER SMOKED CIGARETTES ON A DAILY BASIS FOR A PERIOD OF 1 YEAR OR LONGER?**

☐ 1 = NO, NEVER
☐ 2 = YES, BUT ONLY IN THE PAST
☐ 3 = YES, CURRENTLY
☐ 4 = PREFER NOT TO ANSWER

MEDICAL HISTORY

27. **WHAT IS YOUR HIV STATUS?**

☐ 1 = NEGATIVE
☐ 2 = POSITIVE
☐ 3 = DON'T KNOW

THINPREP COLLECTION (TO BE FILLED BY THE STUDY STAFF)

28**. CERVICAL CELLS IN PRESERVCYT SOLUTION TAKEN (THINPREP)?**

☐ 1 = NO, REASON WHY ______________________________
☐ 2 = YES

COMMENTS (IF ANY)

THANK YOU SO MUCH FOR YOUR TIME AND YOUR PATIENCE.
YOUR PARTICIPATION IS VERY IMPORTANT TO OUR STUDY.
